# Effect of anterior cervical discectomy and fusion versus laminoplasty on spinocranial angle in patients with multilevel cervical spondylotic myelopathy: A retrospective cohort study

**DOI:** 10.1097/MD.0000000000049714

**Published:** 2026-07-17

**Authors:** Zhen Liu, Ji-Hui Zheng, De-Feng Liu, Dong-Dong Su, Zhi-Guang Sun, Yang Liu, Yao Li, Qing Song

**Affiliations:** a Department of Spinal Surgery, Hebei Province Cangzhou Hospital of Integrated Traditional and Western Medicine, Cangzhou, PR China; b Department of Orthopedics, Hebei Key Laboratory of Integrated Traditional and Western Medicine in Osteoarthrosis Research, Cangzhou, PR China.

**Keywords:** anterior cervical discectomy and fusion, laminoplasty, multilevel cervical spondylotic myelopathy, spinocranial angle

## Abstract

This study compared anterior and posterior surgeries regarding sagittal spinocranial angle (SCA) and other sagittal balance metrics, analyzed correlations between radiographic changes and clinical outcomes, and identified the superior surgical strategy for multilevel cervical spondylotic myelopathy (MCSM). This retrospective cohort enrolled 51 patients receiving anterior cervical discectomy and fusion (ACDF) and 69 undergoing posterior laminoplasty (LP) between 2014 and 2021, with a minimum 24-month follow-up. Serial radiography, CT, and MRI measured SCA, surrogate C7 slope (for unavailable T1 slope), C2–C7 Cobb angle (CA), cSVA, and T1sCA at preoperation, early postoperation (5–7 days), and final follow-up. Clinical endpoints included JOA score, neurological recovery rate (RR), NDI, and SF-36. Multivariate regression and linear mixed-effects models adjusted for age, gender, BMI, baseline scores, and operative segments to clarify the independent impacts of surgery and sagittal parameter shifts on clinical results. Both procedures significantly improved all quality-of-life metrics (*P* < .05). ACDF yielded superior final RR (50.50 ± 24.71% vs 44.98 ± 17.77%, *P* = .026) and lower NDI (12.90 ± 4.15 vs 14.97 ± 3.52, *P* = .009). At final follow-up, ACDF presented larger C7 slope and CA (all *P* ≤ .047), alongside reduced SCA, T1sCA and cSVA (all *P* < .001). Multivariate analyses confirmed ACDF independently predicted lower follow-up NDI (β = −2.31, *P* = .009), while elevated ΔSCA independently aggravated NDI (β = 0.38, *P* = .002); neither ACDF nor ΔSCA independently correlated with JOA RR (all *P* > .05). ACDF independently drove postoperative SCA reduction (β = −11.52, *P* < .001). Relative to LP, ACDF reduces postoperative SCA and improves NDI and neurological recovery. Constrained by its retrospective nonrandomized design, these findings await prospective randomized verification; surgical selection should be individualized per patient pathology and sagittal balance.

## 1. Introduction

Multilevel cervical spondylotic myelopathy (MCSM) refers to a type of cervical spondylosis with multiple (≥3) segments of continuous or discontinuous cervical vertebral body posterior marginal bone hyperplasia, osteophyte formation, intervertebral disc degeneration, protrusion, and other pathological changes that cause multiple levels of compression in front of the spinal cord or dural sac and corresponding clinical manifestations. In general, surgical approaches can be divided into anterior, posterior, and anterior and posterior cervical canal decompression approaches. Each approach is supplemented by additional fusion. The anterior approach typically comprises anterior cervical discectomy and fusion (ACDF) and anterior cervical corpectomy with fusion, whereas laminectomy in the presence and/or absence of instrumentation and/or laminoplasty is included in the posterior approach.^[1,2]^ In clinical practice, the most highly regarded procedures are undoubtedly ACDF and laminoplasty (LP). Both ACDF and LP improve health-related outcomes.^[3-8]^ However, the best approach to achieve decompression and correct deformity for MCSM remains controversia.^[9]^ In the literature, changes in sagittal parameters are closely related to the quality of life of patients after cervical surgery.^[10]^ Recent reports have revealed 3 critical sagittal balance parameters widely recognized for cervical balance – the T1-slope (T1s), C2–C7 sagittal vertical axis (cSVA), and spinocranial angle (SCA) – for which postoperative changes are the focus of future research.^[11]^ SCA, as a new parameter to measure sagittal balance, is the angle between a line from the sella turcica center and C7 endplate and the C7 plateau line and usually fluctuates within a certain range (83 ± 9°) among the normal population. It not only considers the cervical base but also the weight of the head. The advantage of SCA lies in that it takes into account both the cervical base inclination and the head weight’s impact on balance, and is directly related to the horizontal fixation function. Studies have shown that SCA is significantly correlated with the C7 anterior curvature angle and the C2–C7 Cobb angle.^[12]^ Although SCA is of great significance for sagittal plane balance, current research on the changes of SCA in MCSM surgery (especially in the comparison of ACDF and LP) and its impact on clinical outcomes is still limited. Moreover, ACDF (direct anterior decompression + fusion) and LP (indirect posterior decompression + non-fusion) have fundamental differences in biomechanical characteristics and indications. Comparing the effects of these 2 surgical procedures on sagittal plane balance is of great clinical significance for providing individualized surgical decisions for different patients. This research report follows the STROBE observational study statement and combines the recommendations of the clinical practice guidelines for the management of degenerative cervical spinal cord disease.^[13,14]^ Therefore, this study aims to fill this knowledge gap, systematically evaluate the effects of ACDF and LP on SCA and other sagittal plane parameters, and analyze the association between these changes and clinical outcomes.

## 2. Materials and methods

### 2.1. Patient population

This study was approved by the Ethics Committee of Cangzhou Integrated Traditional Chinese and Western Medicine Hospital in Hebei Province. Given the retrospective nature of this study, the ethics committee waived the requirement for written informed consent. This study conducted a retrospective analysis of MCSM patients who underwent ACDF and LP by the same surgeon from January 2014 to January 2021.

Inclusion criteria: Clinically diagnosed with cervical spondylotic myelopathy and MRI showing compression of 3 or more vertebral segments; follow-up period of no <24 months, with complete postoperative and follow-up/review data; and both surgical methods were applicable to the patients, and the specific contents of the 2 surgeries were explained to the patients in detail; ultimately, the patients chose the surgical method they received.

**Figure 1.**
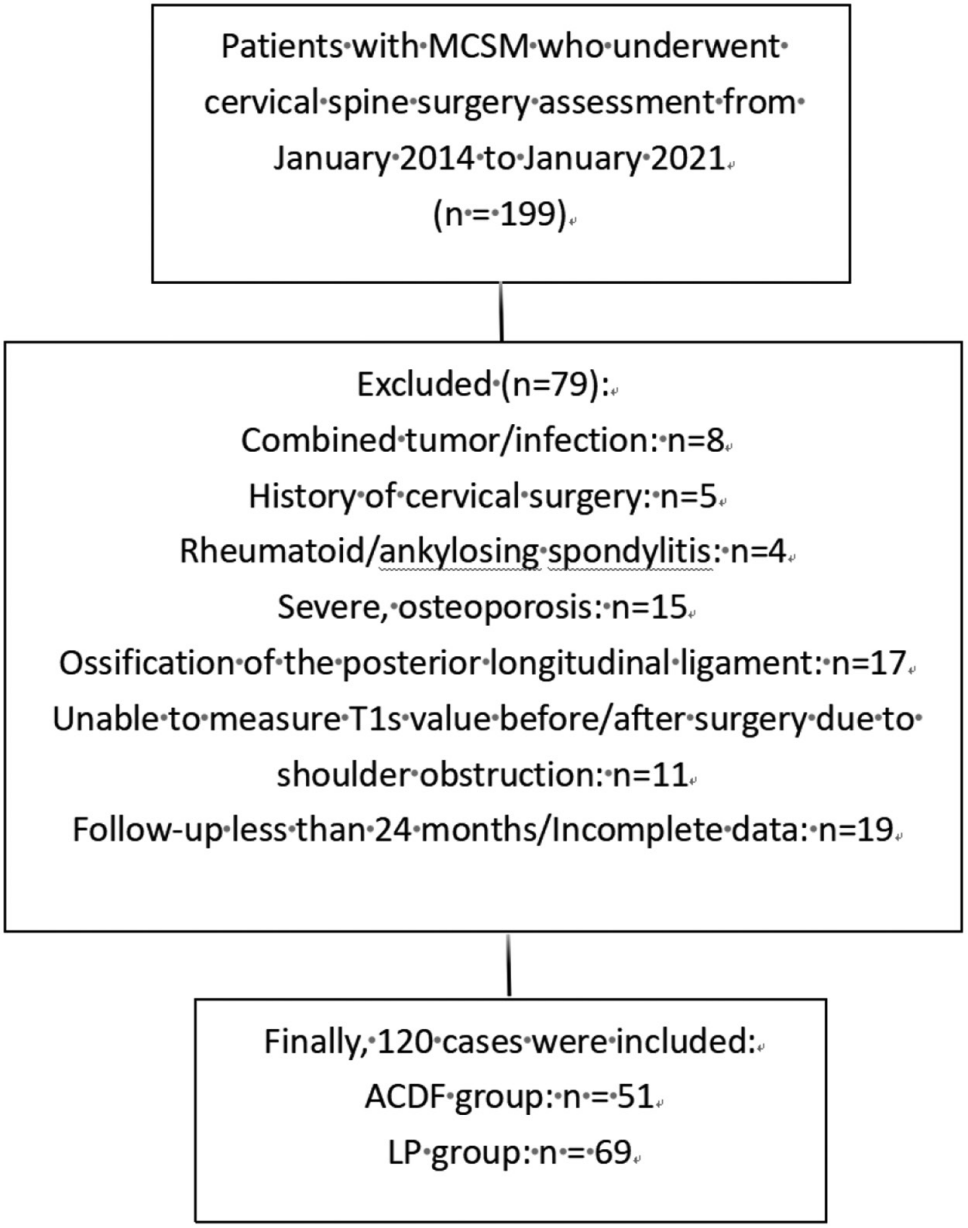
Flow chart. ACDF = anterior cervical discectomy and fusion, LP = laminoplasty, MCSM = multilevel cervical spondylotic myelopathy.

Exclusion criteria: Complicated by trauma, tumor, or infection; history of cervical surgery; rheumatoid arthritis or ankylosing spondylitis; complicated by severe osteoporosis; complicated by posterior longitudinal ligament ossification; and unable to measure T1s value before and after surgery (Fig. 1).

Patients must simultaneously meet the following conditions: MRI indicates spinal cord compression from the front (disc/bone spur) but without significant posterior OPLL compression; preoperative anteroposterior lateral x-ray films without severe local kyphosis deformity (C2–C7 Cobb angle > −10°); compression segments are 3 or 4; no severe comorbidities (such as American Society of Anesthesiologists classification ≥ III) that make them intolerant to either anterior or posterior surgery; no clear cervical segmental instability (displacement < 3.5mm in dynamic position x-ray films); and no severe axial neck pain (visual analogue scale < 7 points).

### 2.2. Surgical technique

*ACDF group*: The Smith–Robinson technique^[15]^ was employed. The surgical segments were determined based on the extent of spinal cord compression. After thorough decompression of the affected segment, a PEEK fusion device with local autologous bone (prepared from the endplate material) was implanted, and anterior plate screws were used for internal fixation.^[16]^

*LP group*: The standard laminoplasty procedure^[17]^ was adopted. The patient was in the prone position, and after anesthesia, the skin, subcutaneous tissue, and fascia were incised, and the bilateral paravertebral muscles were dissected to expose the C3 to C7 laminae. Slots were made at the junction of the facet joints of the bilateral laminae, and the laminae were opened to 1 side and fixed with a micro titanium plate to maintain the expanded state of the spinal canal. The decompression range should cover at least the C3 to C6 segments and can be extended to C7 depending on the degree of compression.

### 2.3. Radiographic analysis

**Figure 2.**
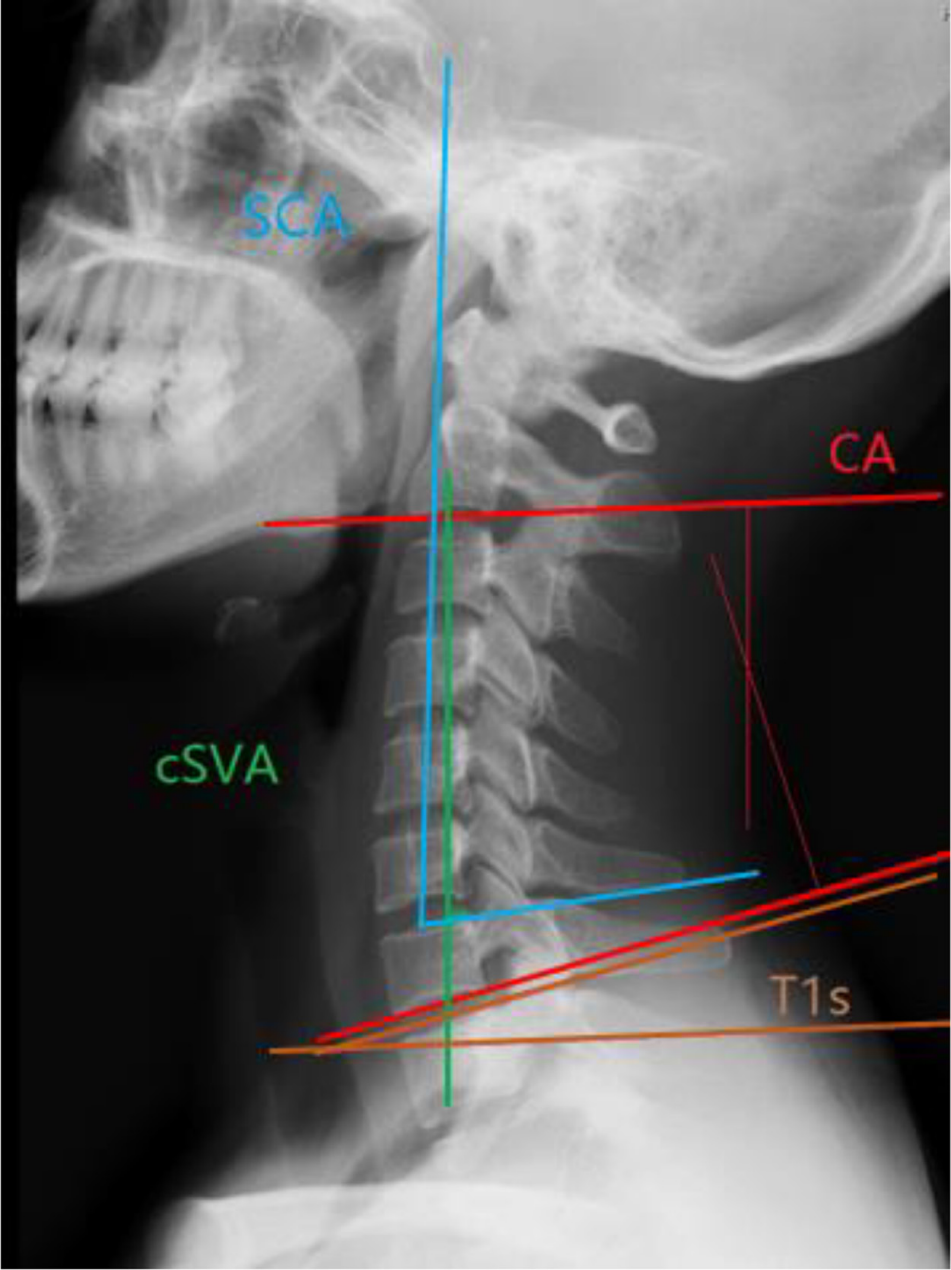
Spinocranial angle (SCA): angle between the C7 slope and a straight line joining the middle of the C7 endplate and the middle of the sella turcica. T1 slope (T1s): angle between a horizontal line and the superior endplate of T1 or C7. C2–C7 lordosis (CA): angle between the lower plate of C2 and the lower plate of C7. C2–C7 SVA (cSVA): distance from the posterior, superior corner of C7 to the plumbline from the centroid of C2. CA = C2–C7 Cobb angle, cSVA = C2–C7 sagittal vertical axis, SCA = spinocranial angle, T1s = T1 slope.

All sagittal plane balance parameters were measured by a spinal surgeon who was not involved in the patient’s clinical treatment. The surgeon was completely unaware of the surgical method and timing (preoperative, postoperative, or follow-up). Each parameter was measured 3 times, and the average value was taken. The diagnosis of MCSM was confirmed through MRI and CT before the surgery. The measurement data of sagittal plane balance parameters (SCA, cSVA, and T1sCA) all came from the cervical neutral lateral x-ray films taken before the surgery, at the time of discharge, on the fifth to seventh day after the surgery, and at the final follow-up (at least 24 months later; Fig. 2).

Due to the obstruction of the scapulae and ribs, the superior endplate of T1 on the x-ray films often cannot be clearly displayed. Therefore, in this study, the slope of the superior endplate of C7 was uniformly measured as the C7 slope and used as a surrogate for T1s in all analyses. The specific parameter definitions are as follows:

SCA: The angle between the tangent of the superior endplate of the C7 vertebra and the line connecting the midpoint of the superior endplate of the C7 vertebra and the center point of the sella turcica. C7 slope (replacing T1s): The angle between the tangent of the superior endplate of the C7 vertebra and the horizontal line. CA (C2–C7 Cobb angle): The angle between the tangent of the lower endplate of the C2 vertebra and the tangent of the lower endplate of the C7 vertebra. Lordosis is positive, kyphosis is negative. cSVA (C2–C7 sagittal plane vertical axis): The horizontal distance (mm) between the posterior upper angle of the C7 vertebra and the vertical line passing through the geometric center of the C2 vertebra. T1sCA: The C7 slope minus CA.

Randomly selected 20 patients’ preoperative and postoperative x-ray films were measured by the same measurer at intervals of 2 weeks for all parameters, and the intraclass correlation coefficient (ICC) was calculated. The results showed that the ICCs of SCA, C7 slope, and CA were all >0.90 (95% CI: 0.85–0.95), indicating excellent measurement reliability.

### 2.4. Clinical outcome assessment

Collect health-related outcome indicators of the patients before surgery, after surgery, and at the last follow-up, including: Japanese Orthopaedic Association (JOA) score (0–17 points), recovery rate (RR) = (JOA after surgery − JOA before surgery)/ (17 − JOA before surgery) × 100%, neck dysfunction index (NDI; 0–50 points), and quality of life scale SF-36 score (0–100 points). At the same time, record the operation time, blood loss volume, hospital stay, and complications.

### 2.5. Statistical analysis

The analysis was conducted using SPSS 26.0 (version 26.0; SPSS Inc., Chicago) and R 4.1 software (version 4.1; R Core Team, The R Foundation for Statistical Computing). Continuous variables were expressed as mean ± standard deviation. A normality test was performed using the Shapiro–Wilk test, and homogeneity of variance was tested using the Levene test. Comparisons between groups with normal distribution and homogeneous variance were conducted using the independent sample *t* test, while intragroup comparisons were performed using the paired *t* test; those that did not meet these criteria were analyzed using the Mann–Whitney *U* test or the Wilcoxon signed-rank test. Categorical variables (gender, complications) were analyzed using the chi-square test. Multivariate analysis: To control for confounding bias, a multiple linear regression model was used to analyze the independent association between the surgical method (ACDF vs LP) and the primary outcome indicators (NDI at the last follow-up, JOA RR, SCA change). The covariates adjusted in the model included age, gender, BMI, baseline JOA score, baseline NDI score, baseline SCA, baseline C7 slope, and the number of surgical segments (continuous variable). Results were reported as the adjusted β coefficient and its 95% confidence interval (CI). Repeated measures analysis: A linear mixed-effects model (LMM) was used to analyze the trends of sagittal plane parameters and clinical outcomes over time (preoperatively, postoperatively, and at the last follow-up) and the significance of the interaction between groups (ACDF vs LP) and time. The patient ID was used as the random intercept in the model. All statistical tests were 2-sided, and the significance level was set at α = 0.05.

## 3. Results

### 3.1. Demographic data

**Figure 3.**
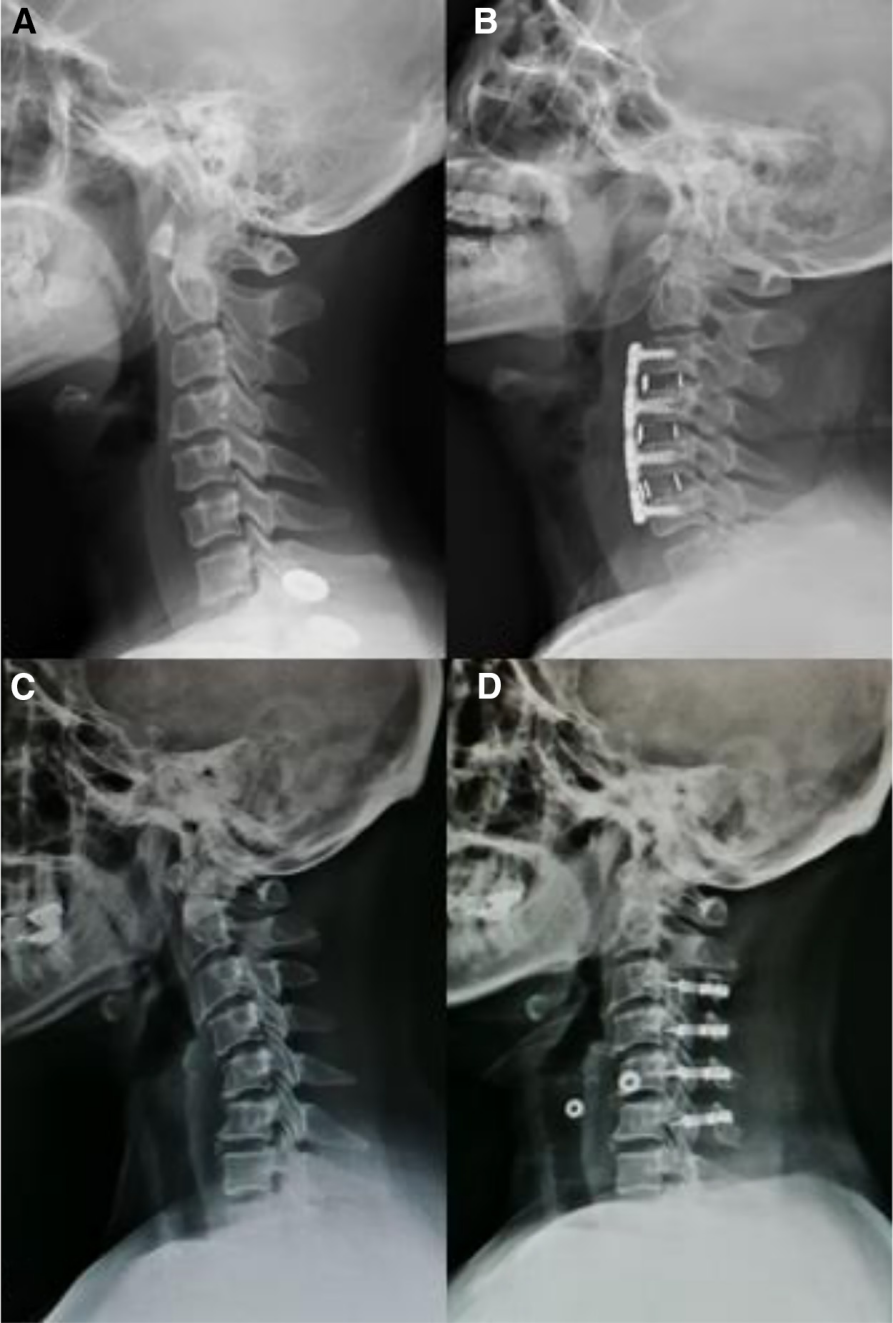
Multilevel anterior cervical discectomy and fusion (ACDF) and laminoplasty (LP) were performed to release the compression. Lateral x-rays of the cervical spine were taken in patients with multilevel cervical spondylotic myelopathy (MCSM) under ACDF preoperatively (A), at the 2-year follow-up visit (B), in patients with MCSM under LP preoperatively (C), and at the 2-year follow-up visit (D). ACDF = anterior cervical discectomy and fusion, LP = laminoplasty, MCSM = multilevel cervical spondylotic myelopathy.

A total of 120 subjects (M = 64, F = 56) who matched conditions were identified. Among them, 51 patients had undergone ACDF (Fig. 3A, B), and 69 patients had undergone LP (Fig. 3C, D). There were no significant differences in age, gender, BMI, or preoperative JOA and NDI scores between the 2 groups (*P* > .05), indicating good baseline comparability (Table 1). The number of surgical segments in the ACDF group and the LP group was 3.04 ± 0.20 segments and 3.38 ± 0.64 segments, respectively (*P* < .001). The operation time of the ACDF group and the LP group was 135.5 ± 28.9 minutes and 123.0 ± 29.2 minutes, respectively (*P* = .011); the blood loss was 145.5 ± 34.0 mL and 188.8 ± 61.5 mL, respectively (*P* < .001); the hospital stay was 5.8 ± 1.7 days and 8.3 ± 1.6 days, respectively (*P* < .001). The specific data are shown in Table 1. In the ACDF group, the most common fusion segments were C4–6 (18 cases, 35.3%), followed by C3–5 (15 cases, 29.4%) and C4–7 (12 cases, 23.5%); in the LP group, the most common decompression segments were C3–6 (32 cases, 46.4%), followed by C3–7 (25 cases, 36.2%).

**Table 1:** Comparison of patient characteristics.

	ACDF (n = 51)	LP (n = 69)	*P*-value
Number of patients	51	69	
Age (yr)	57.08 ± 7.15	58.19 ± 8.74	.289
Sex (male/female)	29/22	35/34	.505
body mass index (BMI)	24.60 ± 5.68	24.43 ± 5.06	.803
Operation time (min)	135.49 ± 28.94	123.04 ± 29.17	.011
Number of complications	5	8	.755
Length of hospitalization	5.82 ± 1.67	8.26 ± 1.56	<.001
Number of operative segments	3.04 ± 0.20	3.38 ± 0.64	<.001
Three sections	32 (62.75%)	49 (71.01%)	.339
Four sections	19 (37.25%)	20 (28.99%)	
Blood loss	145.49 ± 33.96	188.84 ± 61.47	<.001

ACDF = anterior cervical discectomy and fusion, BMI = body mass index, LP = laminoplasty.

### 3.2. Comparison of sagittal plane parameters

*Group comparison*: Before surgery, there were no significant differences in all sagittal plane parameters between the 2 groups. At the postoperative and final follow-up, the C7 slope and CA in the ACDF group were significantly higher than those in the LP group, while SCA, T1sCA, and cSVA were significantly lower than those in the LP group (Table 2). The linear mixed-effects model showed that there was a significant interaction between the surgical method and time on SCA (*P* < .001), indicating that the trends of SCA change over time were different in the 2 groups.

**Table 2:** Comparison of cervical sagittal parameters.

	ACDF (n = 51)	LP (n = 69)	Mean difference (95% CI)	*P*-value
T1s (°)				
Pre	25.65 ± 7.86	25.67 ± 8.13	−0.02 (−3.01 to 2.97)	.909
Post	27.35 ± 7.74	24.30 ± 6.86	3.05 (0.09 to 6.01)	.043
F/U	27.31 ± 7.87	24.17 ± 6.28	3.14 (0.04 to 6.24)	.047
△T1s (°) (pre vs post)	1.70 ± 2.58	−1.37 ± 5.08	3.07 (1.66 to 4.48)	<.001
△T1s (°) (pre vs F/U)	1.66 ± 2.41	−1.50 ± 3.98	3.16 (2.09 to 4.23)	<.001
Pre vs F/U	<0.001	0.004		
CA (°)				
Preoperative	14.45 ± 8.71	14.15 ± 8.28	0.30 (−2.79 to 3.39)	.851
Post	18.94 ± 8.15	12.16 ± 8.27	6.78 (3.82 to 9.74)	<.001
F/U	20.91 ± 7.62	11.27 ± 7.78	9.64 (6.74 to 12.54)	<.001
△CA (°) (pre vs post)	4.49 ± 6.54	−1.99 ± 2.27	6.48 (4.82 to 8.14)	<.001
△CA (°) (pre vs F/U)	6.46 ± 6.24	−2.89 ± 3.19	9.35 (7.65 to 11.05)	<.001
Pre vs F/U	<0.001	<0.001		
cSVA (mm)				
Pre	18.50 ± 6.30	20.21 ± 9.87	−1.71 (−4.73 to 1.31)	.249
Post	25.90 ± 7.34	30.02 ± 7.57	−4.12 (−7.13 to −1.11)	.008
F/U 2y	26.45 ± 12.26	34.89 ± 8.64	−8.44 (−12.87 to −4.01)	<.001
△cSVA (mm) (pre vs post)	7.40 ± 6.13	9.81 ± 8.14	−2.41 (−4.99 to 0.17)	.068
△cSVA (mm) (pre vs F/U)	7.95 ± 12.61	14.68 ± 10.57	−6.73 (−10.95 to −2.51)	.002
Pre vs F/U	<0.001	<0.001		
SCA (°)				
Pre	81.68 ± 9.76	79.46 ± 9.82	2.22 (−1.42 to 5.86)	.207
Post	78.10 ± 9.37	84.86 ± 8.69	−6.76 (−10.18 to −3.34)	<.001
F/U 2y	77.12 ± 9.31	86.95 ± 9.15	−9.83 (−13.20 to −6.46)	<.001
△SCA (°) (pre vs post)	−3.58 ± 5.07	5.39 ± 9.21	−8.97 (−11.32 to −6.62)	<.001
△SCA (°) (pre vs F/U)	−4.56 ± 5.16	7.49 ± 9.74	−12.05 (−14.68 to −9.42)	<.001
Pre vs F/U	<0.001	<0.001		
T1sCA (°)				
Pre	11.20 ± 6.64	11.51 ± 11.26	−0.31 (−3.80 to 3.18)	.962
Post	8.41 ± 8.43	12.14 ± 10.07	−3.73 (−7.17 to −0.29)	.034
F/U 2y	6.40 ± 7.62	12.90 ± 8.86	−6.50 (−9.55 to −3.45)	<.001
△T1sCA (°) (pre vs post)	−2.79 ± 7.26	0.63 ± 5.54	−3.42 (−5.74 to −1.10)	.010
△T1sCA (°) (pre vs F/U)	−4.80 ± 7.08	1.38 ± 5.07	−6.18 (−8.41 to −3.95)	<.001
Pre vs F/U	<0.001	0.014		

ACDF = anterior cervical discectomy and fusion, CA = C2–7 lordosis angle, CI = confidence interval, cSVA = C2–7 sagittal vertical axis, F/U = follow-up, LP = laminoplasty, pre = preoperative, post = postoperative, SCA = spinocranial angle, T1s = T1-slope (measured as C7 slope as a surrogate due to shoulder shadowing, see Section 2), T1sCA = T1-slope (C7 slope) minus C2–7 lordosis angle, △(pre vs F/U) = follow-up value − preoperative value.

95% confidence intervals for the mean Δ values (Pre vs F/U):

△T1s: ACDF 1.66° (95% CI: 1.00–2.32), LP −1.50° (95% CI: −2.44 to −0.56);

△CA: ACDF 6.46° (95% CI: 4.75–8.17), LP −2.89° (95% CI: −3.64 to −2.14);

△cSVA: ACDF 7.95 mm (95% CI: 4.49–11.41), LP 14.68 mm (95% CI: 12.19–17.17);

△SCA: ACDF −4.56° (95% CI: −5.98 to −3.14), LP 7.49° (95% CI: 5.19–9.79);

△T1sCA: ACDF −4.80° (95% CI: −6.74 to −2.86), LP 1.38° (95% CI: 0.18–2.58).

*Group changes*: In the ACDF group, compared with preoperative values, at the last follow-up, the C7 slope, CA, and cSVA all significantly increased (all *P* < .001), while SCA and T1sCA both significantly decreased (all *P* < .001). In the LP group, compared with preoperative values, at the last follow-up, cSVA, T1sCA, and SCA all significantly increased (*P* < .05), while the C7 slope and CA both significantly decreased (*P* < .01). Specific data are shown in Table 2.

### 3.3. Clinical outcomes

Both surgical methods significantly improved the JOA, NDI, and SF-36 scores (Table 3). The last follow-up JOA RR in the ACDF group was significantly higher than that in the LP group (50.5% vs 45.0%, mean difference 5.52%, 95% CI: 0.67%–10.37%, *P* = .026). The last follow-up NDI score in the ACDF group was significantly lower than that in the LP group (12.90 vs 14.97, mean difference −2.07, 95% CI: −3.59 to −0.55, *P* = .009). After multivariate regression analysis adjusted for age, gender, BMI, baseline JOA/NDI, number of surgical segments, and preoperative SCA, the results showed that ACDF remained an independent related factor for lower NDI at the last follow-up (β = −2.31, 95% CI: −4.02 to −0.60, *P* = .009). In addition, when including the change in SCA (△SCA) as a continuous variable in the model, for every 1° increase in △SCA, the follow-up NDI increased by 0.38 points (β = 0.38, 95% CI: 0.15–0.61, *P* = .002), indicating that the increase in SCA was independently associated with worse cervical function. The adjusted *R*^2^ of the multivariate linear regression model was 0.42, indicating that the model explained 42% of the variation in NDI. There was no significant statistical difference in the degree of improvement of JOA and SF-36 scores between the 2 groups.

**Table 3:** Quality of life parameters.

	ACDF (n = 51)	LP (n = 69)	Mean difference (95% CI)	*P*-value
JOA				
Pre	10.10 ± 1.60	9.97 ± 2.16	0.13 (−0.60 to 0.86)	.809
Post	13.14 ± 1.44	12.54 ± 1.53	0.60 (−0.02 to 1.22)	.060
F/U 2y	13.78 ± 1.19	13.38 ± 1.00	0.40 (−0.02 to 0.82)	.063
△JOA (pre vs post)	3.04 ± 1.51	2.57 ± 1.64	0.47 (−0.02 to 0.96)	.057
△JOA (pre vs F/U)	3.69 ± 1.77	3.41 ± 1.95	0.28 (−0.42 to 0.98)	.214
Pre vs F/U	<0.001	<0.001		
RR (pre vs post)	42.80% ± 22.12%	34.16% ± 20.40%	8.64 (2.16 to 15.12)	.009
RR (pre vs F/U)	50.50%±24.71%	44.98%±17.77%	5.52 (0.67 to 10.37)	.026
NDI				
Pre	20.98 ± 5.65	21.45 ± 5.14	−0.47 (−2.44 to 1.50)	.504
Post	14.35 ± 4.17	15.88 ± 3.66	−1.53 (−2.97 to −0.09)	.035
F/U 2y	12.90 ± 4.15	14.97 ± 3.52	−2.07 (−3.59 to −0.55)	.009
△NDI (pre vs post)	−6.63 ± 2.49	−5.57 ± 3.48	−1.06 (−2.15 to 0.03)	.058
△NDI (pre vs F/U)	−8.08 ± 3.01	−6.48 ± 3.59	−1.60 (−2.82 to −0.38)	.011
Pre vs F/U	<0.001	<0.001		
SF-36				
Pre	43.73 ± 6.23	44.55 ± 6.69	−0.82 (−3.21 to 1.57)	.496
Post	52.63 ± 7.08	52.96 ± 8.40	−0.33 (−3.16 to 2.50)	.667
F/U 2y	53.61 ± 7.34	53.49 ± 7.87	0.12 (−2.73 to 2.97)	.935
△SF-36 (pre vs post)	8.90 ± 4.39	8.41 ± 4.74	0.49 (−1.12 to 2.10)	.420
△SF-36 (pre vs F/U)	9.88 ± 4.04	8.94 ± 4.56	0.94 (−0.67 to 2.55)	.146
Pre vs F/U	<0.001	<0.001		

The formula for RR is provided in Section 2.4 (clinical outcome assessment).

95% confidence intervals for the mean Δ values (pre vs F/U):

△JOA: ACDF 3.69 (95% CI: 3.20–4.18), LP 3.41 (95% CI: 2.95–3.87);

△NDI: ACDF −8.08 (95% CI: −8.91 to −7.25), LP −6.48 (95% CI: −7.33 to −5.63);

△SF-36: ACDF, 9.88 (95% CI: 8.77–10.99); LP, 8.94 (95% CI: 7.86–10.02).

ACDF = anterior cervical discectomy and fusion, CI = confidence interval, F/U = follow-up, JOA = Japanese Orthopaedic Association, LP = laminoplasty, NDI = Neck Disability Index, pre = preoperative, post = postoperative, RR = recovery rate.

### 3.4. Complications

In the ACDF group, 2 cases (3.9%) experienced aggravated degeneration of the adjacent segment, and 3 cases (5.9%) had mild dysphagia, both of which resolved within 6 months. In the LP group, 4 cases (5.8%) suffered from C5 nerve root palsy, and 2 of them recovered during the follow-up period; 4 cases (5.8%) had axial symptoms. There was no significant difference in the total complication rate between the 2 groups (9.8% vs 11.6%, χ^2^ = 0.10, *P* = .755).

## 4. Discussion

Both ACDF and LP are classic surgical approaches for treating MCSM. ACDF can directly relieve anterior compression on the spinal cord, restore the height of the intervertebral space and cervical lordosis, provide immediate stability, and eliminate dynamic compression.^[18]^ LP, on the other hand, achieves indirect spinal cord decompression by expanding the posterior space of the spinal canal while preserving cervical mobility. However, it may carry risks such as axial pain, C5 nerve root palsy, and progression of kyphosis.^[19]^ This retrospective cohort study, after controlling for multiple confounding factors, systematically compared the effects of the 2 surgical methods on the novel sagittal plane balance parameter SCA and their associations with clinical outcomes.

### 4.1. Comparison of health-related outcomes

Similar to published conclusions, the results revealed that both ACDF and LP enhanced patient function. At the same time, RR JOA in ACDF was significantly higher than that in LP at post (*P* = .009) and F/U (*P* = .026), indicating that ACDF may have better neurological recovery. Regarding NDI, ACDF also performed better than LP at post (*P* = .035) and F/U (*P* = .009). More interestingly, although the △NDI (pre vs post) of the 2 groups did not show a significant difference (*P* = .058), △NDI (pre vs F/U) in ACDF was greater than that in LP, which showed a significant difference (*P* = .011), indicating that the long-term improvement of neck pain symptoms in ACDF may be better. The reason might be that patients who received ACDF treatment underwent a more appropriate fusion procedure; excessive spinocranial angle (SCA) can pull on various muscles attached to the neck, thereby triggering the neck’s pain threshold.

Additionally, blood loss and hospital stay in ACDF were reduced compared with those in LP, revealing significant differences (BL: *P* < .001; LOH: *P* < .001). This finding may reflect that ACDF has better tolerance than LP, where extensive internal muscle dissection and a larger area exposed to the outside environment lead to longer hospital stays and greater postoperative discomfort, consistent with that reported in a published review.^[20]^ However, concerning operation time, our study showed that ACDF takes significantly longer than LP (*P* = .011). No significant difference was found in the complication rate between ACDF and LP (*P* = .796). In contrast, the mechanism of increased SCA after LP surgery is the opposite: destruction of the posterior structures leads to a tendency for the cervical spine to become kyphotic (CA decreased by 2.89°), and to maintain a horizontal line of sight, the body compensates by increasing SCA (in this study, SCA increased by 7.49° in the LP group), which is consistent with the positive correlation between SCA and CA reported by Le Huec et al.^[12]^

### 4.2. Comparison of different sagittal parameters

Le Huec et al^[12]^ proposed a new parameter, SCA22, for measuring sagittal plane balance based on a prospective study of the general population. SCA corresponds to the angle between the tangent of the upper C7 platform and the line connecting the middle part of the upper C7 platform to the center of the sella turcica. This parameter takes into account not only the angle of the cervical base but also the weight of the head. In asymptomatic volunteers, this angle should remain within the normal range (83° ± 9°). The SCA indicator shows that the head maintains a horizontal gaze position and is negatively correlated with the C7 segment and positively correlated with the CA15 segment. Previous studies have shown that SCA can be regarded as a key parameter for predicting sagittal plane balance.^[21–24]^ This may be because an increase in SCA is accompanied by a decrease in the slope of the C7 segment and the disappearance of lumbar lordosis,^[10,25]^ thereby affecting horizontal visual function. Therefore, patients may compensate for the cervical balance state by reducing the T1s value, causing various muscles attached to the neck to be stretched, and subsequently triggering the pain threshold and increasing energy consumption. Wang et al^[23]^ reported that patients with a lower SCA were more likely to experience greater sagittal plane changes after laminoplasty and could maintain better sagittal plane balance, thereby achieving a more ideal surgical outcome. In this study, the postoperative and follow-up SCA values of the LP group were significantly higher than those of the ACDF group (postoperative: *P* < .001; follow-up: *P* < .001). The various indicators of the LP group at postoperative and follow-up were also significantly better than those of the ACDF group (postoperative: *P* < .001; follow-up: *P* < .001), indicating that the LP corresponds to a larger SCA angle. Therefore, when SCA is large, LP may not be an ideal choice; likewise, when SCA is below the normal angle, the LP surgical method can also be considered. The results of this study are consistent with previous research findings.^[26]^ The mechanism for the decrease in SCA after ACDF surgery can be explained as follows: the implantation of the fusion device expands the intervertebral space, increases the height of the anterior column, and causes an increase in the anterior curvature of the surgical segment, thereby increasing the overall cervical lordosis angle (CA; in this study, the CA of the ACDF group increased by 6.46°, *P* < .001). The increase in cervical lordosis causes a relative decrease in the slope of the C7 upper endplate (C7 slope). When the body compensates for the backward tilt to maintain a horizontal line of sight, the angle between the line connecting the sella turcica and the midpoint of the C7 upper endplate and the C7 slope (i.e., SCA) decreases. Conversely, after LP surgery, the posterior structure is destroyed, and the cervical spine tends to develop kyphosis (CA decreased by 2.89°, *P* < .001), and the body compensates by increasing SCA (in this study, the SCA of the LP group increased by 7.49°, *P* < .001). According to previous literature.^[25,27]^ After the patient undergoes LP, the cervical lordosis angle (CA) will further decrease. This may be because the further reduction of CA will affect horizontal vision. Therefore, patients may attempt to restore horizontal vision by reducing the T1s value. T1s value and CA are both key parameters for cervical sagittal plane balance, and they support the cervical spine at the anatomical and biomechanical levels through the connection of various muscles. This study shows that compared with LP, the T1s value of patients in the ACDF group is higher (*P* = .043), and the CA after surgery is higher (*P* < .001). The F/U T1s values slightly recovered between the 2 groups; however, the CA at the F/U level continued to improve after surgery. A previous investigation found that T1s values > 40° are associated with positive sagittal plane imbalance.^[28]^ The reasons may be as follows: patients with higher T1s values require more muscle activity, and an increase in T1s value significantly enhances CA. This result indicates that the muscles at the back of the neck are more shortened.^[29]^ Finally, the muscle contraction state may lead to the appearance of painful trigger points. However, in this study, the T1s values of the posterior group and the anterior group rarely exceeded 40 degrees, indicating that the increase was relatively limited. While a moderate increase in T1s values implies a more balanced spinal state, which is closely related to the occurrence of axial symptoms after surgery.^[30]^ These results are consistent with previous research conclusions. Regarding cervical lordosis (CA), postoperative lordosis can bring better therapeutic effects, and the possible reason is that the energy consumption of the neck muscles and the ligaments around the cervical vertebrae is reduced.^[31–36]^ In this study, the ACDF procedure was associated with a significant increase in the lordosis angle on the anterior-posterior T1-weighted image (*P* < .001), while the LP showed the opposite trend. This trend is consistent with the literature reports: loss of lordosis is associated with adverse health outcomes.^[4,25,37–39,40]^ This study suggests that ACDF may be more beneficial for correcting kyphosis and maintaining postoperative lordosis than LP (CA increased by 6.46° in the ACDF group and decreased by 2.89° in the LP group), but this conclusion needs to be confirmed by prospective studies. This indicates that patients tend to tilt the cervical spine forward after ACDF surgery, thereby achieving a better health prognosis.

cSVA is another key parameter for predicting balanced outcomes. Some scholars have confirmed that cSVA is negatively correlated with the health-related outcomes of patients with MCSM. Although the cSVA of both groups increased significantly (*P* < .001), the values measured at the post and F/U time points in the LP group were significantly higher than those in the ACDF group (post: *P* = .008; F/U: *P* < .001; Table 2), which is consistent with the results of previous literature.^[40]^ It is noteworthy that at the F/U time point, the △cSVA of the LP group was significantly higher than that of the ACDF group (*P* = .002); while at the post time point, this difference was not statistically significant (*P* = .068), indicating that the cSVA of the LP group may gradually increase over time and has a poorer prognosis.

Furthermore, T1sCA refers to the additional angle required for the cervical spine to return to the horizontal plane after compensating for T1S through posterior curvature. This indicator has been used to assess cervical spine alignment.^[41,42]^ Studies have found significant differences between the ACDF group and the LP group in post-T1sCA (*P* = .034) and F/U-T1sCA (*P* < .001), indicating that the post-T1sCA and F/U-T1sCA values of the ACDF group are smaller and the functional recovery is better. Patients with lower T1sCA values may achieve more significant functional recovery. The reasons for this may be as follows: when T1sCA decreases, most suboccipital muscles and neck muscles remain in a relaxed state, significantly alleviating postoperative cervical pain. In addition, when the cervical spine tilts backward, vision can be restored more easily, and cervical balance can be more easily maintained.^[43]^ Therefore, from this perspective, the ACDF surgical method is superior to the LP surgical method.

T1s, as an important parameter for cervical spine sagittal plane balance, has been widely studied and can achieve balanced upright posture and appropriate horizontal gaze angle through adjustment.^[44–46]^ Therefore, more and more researchers have recognized the important role of T1s in cervical spine reference positioning. However, this study found that T1s has 2 limitations in evaluating the changes in sagittal plane alignment: First, T1s does not consider the influence of head weight. When the cervical spine is in flexion or extension, the posterior convexity or anterior convexity force of the spine will act on the cervical spine, causing changes in cervical balance. Second, due to the obstruction of the shoulders and ribs, T1s is difficult to measure accurately on lateral x-ray films. Therefore, SCA has attracted increasing attention from scholars and has been proven to have a significant correlation with T1 signal and CL values. Moreover, the study innovatively proposed the concept of “head offset,” which not only considers the “basic structure” of the cervical spine but also incorporates the weight factor of the head.^[12]^

This study provides different sagittal balance parameters for 2 treatment methods. However, this study has several limitations: It is a retrospective, non-randomized design. Although this study used multivariate regression and mixed-effects models to correct for known confounding factors, it is impossible to completely eliminate unmeasured confounding bias (such as patients’ preferences for surgery, surgeons’ experience tendencies, etc). The surgical method was chosen by the patients themselves rather than randomly assigned, which may lead to selection bias. Due to the limitations of the medical record system, this study was unable to record the specific surgical segment combinations for each patient (such as C3–C5, C3–C6, etc) and could only provide the average number of surgical segments. This limits the assessment of the impact of segment distribution differences on the results. Due to shoulder shadowing, this study used the C7 slope to replace T1s, although the 2 are highly correlated, they are not completely equivalent and may introduce systematic errors. SCA, as a relatively new parameter, has not had its measurement reliability and clinical minimal important difference (MCID) fully established. In addition, although the postoperative x-ray films were uniformly taken before discharge (5–7 days after surgery), the number of surgical segments and the type of internal fixation of different patients may affect the early sagittal plane parameters. Ideally, a more standardized follow-up time point (such as 3 months after surgery) should be used. The surgical method was chosen by the patients themselves rather than randomly assigned. Although this study corrected for known confounding factors, it cannot rule out selection bias due to patient preferences or doctor tendencies. No subgroup analysis was conducted on specific surgical segment distributions (such as C3–C5 vs C3–C7), which may affect the interpretation of the results. This study did not evaluate the minimum clinical important difference (MCID) of SCA, NDI, etc, and the clinical significance of statistical significance needs to be further verified.

Based on the results of this study, for patients with MCSM who require surgical treatment, if imaging shows that spinal cord compression mainly comes from the front, the preoperative cervical lordosis is still acceptable (CA > 0°), and the preoperative SCA is high or normal, ACDF may be a reasonable choice to achieve better sagittal plane balance and recovery of cervical function. For patients with severe posterior ligament ossification and severe spinal canal stenosis but good preoperative lordosis, LP can still be an effective posterior indirect decompression option. The final decision should be made based on individualized selection, considering the source of compression, sagittal plane balance status, patient age, and comorbidities.

## 5. Conclusion

Under the conditions of this retrospective cohort study, compared with laminoplasty, ACDF was associated with lower postoperative SCA values, better NDI, and better nerve function RR. Multivariate analysis suggested that the change in SCA was an independent factor affecting postoperative cervical function. However, given the non-randomized, retrospective design of this study and potential confounding bias, this conclusion needs to be verified by high-quality prospective randomized controlled studies. The choice of surgical method should be based on a comprehensive assessment of the individualized pathological characteristics of the patient (source of compression, sagittal plane balance status).

## Author contributions

**Conceptualization:** Zhen Liu.

**Data curation:** Ji-Hui Zheng, De-Feng Liu, Zhi-Guang Sun, Yang Liu, Qing Song.

**Formal analysis:** Dong-Dong Su, Yao Li.
